# Studying the Efficacy of Psychodrama With the Hermeneutic Single Case Efficacy Design: Results From a Longitudinal Study

**DOI:** 10.3389/fpsyg.2018.01662

**Published:** 2018-09-10

**Authors:** António-José Gonzalez, Paulo Martins, Margarida Pedroso de Lima

**Affiliations:** ^1^ISPA – University Institute of Psychological, Social and Life Sciences, Lisbon, Portugal; ^2^Laboratory of Sport and Exercise Psychology, Faculty of Human Kinetics, University of Lisbon, Cruz Quebrada, Portugal; ^3^Department of Clinical Psychology, Faculty of Psychology and Educational Sciences, University of Coimbra, Coimbra, Portugal

**Keywords:** psychodrama, hermeneutic single case efficacy design, psychotherapy efficacy, spontaneity, single case study

## Abstract

Throughout the last decades, scientific and therapeutic communities have made common efforts to collect reliable information concerning the efficacy of psychotherapies. One of these initiatives has, recently, involved the psychodrama community and its desire to achieve progress in the validation of this therapy. Based on Robert Elliott’s Hermeneutic Single Case Efficacy Design, we followed five participants (three women, two men, aged 27–48 years) of a psychodrama group over the course of their therapeutic process, which ranged from 24 months to 5 years. For the single case study, we selected the participant who had the longest data collecting record, including one follow-up. Participants generally reported improvement in their personal therapeutic goals, decrease in symptoms and life problems, and some showed a marked increase in spontaneity levels. In the single case, these results are confirmed, and following decision criteria it is possible to assert that the participant improved in all the variables assessed and that therapy is the main cause of these changes. Furthermore, the participant frequently rated psychodrama sessions as being helpful and stated they had a transformational impact on his life. This research contributes toward validating psychodrama as an efficient therapeutic method, hopefully stimulating practitioners to integrate therapy and research—which, for years, were considered independent and incompatible—and to facilitate their use in a complementary way.

## Introduction

The field of psychotherapy is nowadays a complex body, where extremely diverse practices and theoretical proposals coexist. Since the very beginning of psychotherapeutic history and tradition, one of the most relevant debates has been on which practices should be considered effective and which should be considered non-effective or even fraudulent. If we go back to the 18th century and remember the polemic with Franz Anton Mesmer’s treatments, based on the pseudo-scientific concept of *animal magnetism* influenced by Newton’s works, we can see that the debate regarding the use of science to both inspire and assess psychotherapeutic practice has existed since the creation of psychotherapy as we know it today. Are specific therapeutic models and schools more efficient than others? Does the person of the therapist make a difference? What is the importance of the client’s characteristics? How should we measure the impacts of treatment? Is psychotherapy, as a practice, becoming more effective in general over the years? What is the role of deliberate practice in the improvement of the therapists? These are just some of the interrogations that researchers in the field have been trying to answer over the last few decades, in an attempt to overcome the fateful words of Eysenck (1952) concerning the ineffectiveness of psychotherapy (for some answers to these questions or more debate, see Elliott and Zucconi, 2006; Castonguay et al., 2010; Norcross and Lambert, 2011; Norcross and Wampold, 2011; Lambert, 2013a,b; Miller et al., 2013; Chow et al., 2015; Wampold and Imel, 2015; Goldberg et al., 2016; Goodyear et al., 2017).

One of the most important issues underlying these debates is the assessment of therapeutic progress. How should we evaluate what is happening in treatment? Should we emulate the medical model of clinical assessment, or should psychotherapy create specific models for this goal? Differently from most medical interventions, the psychotherapeutic format is based on long-term encounters between the people involved, so not only is the “what – if something –changed” an important issue, but the “how did the change occur” becomes essential to the improvement of efficacy of practice. Consequently, the work that sustains Empirically Supported Treatments increased (Sousa, 2017). Having this in mind, mixed models of research, involving both outcome and process measures, were developed. That is the case of the hermeneutic single case efficacy design (HSCED).

This research design was proposed by Robert Elliott, in his 2002 seminal work, and has been used to study the efficacy of several treatments, inspired in different psychotherapeutic models [Carvalho et al., 2008; Elliott et al., 2009, 2011; see Benelli et al. (2015) for a systematic review]. HSCED, an interpretative approach to evaluating treatment causality in single therapy cases, was suggested by Elliott (2002) as an alternative to the randomized clinical trial approach. In the attempt to respond to its limitations concerning the issue of the psychotherapeutic efficacy, a blend of qualitative and quantitative methods from different origins are proposed. We can say that the need to evaluate the causal role of the therapy process stresses the importance of HSCED. This method uses direct and indirect evidences, a rich and comprehensive collection of information about the client’s therapeutic process, and takes in consideration the client as a co-investigator.

In order to build a rich case record and further establish empirical-based evidences, Elliott (2002, 2010) suggests a list of six data collecting strategies: (1) basic data from both client and therapist; (2) quantitative outcome measures, that should be used at beginning, during and end of therapy, and if possible in a follow-up; (3) Client Change Interview (CCI) (see below) in order to assess the client’s view on potential changes throughout the therapeutic process and to establish connections between treatment and changes; (4) weekly simple and easy to answer measure(s) of outcome, usually the Personal Questionnaire (PQ) or another very short instrument; (5) a measure of the client’s impression of the sessions, focused in the significant events that might have occurred, like the Helpful Aspects of Therapy form; and (6) notes from the sessions by the therapist.

Our research aims to study the efficacy of psychodrama as a method of group psychotherapy in which clients are helped to solve their problems not only by speaking about them but by acting them out. As stated by Wieser (2007, p. 271), “Psychodrama as psychotherapy is based on theories of spontaneity, creativity and action. It is probably due to this association that the study of psychodrama’s effectiveness, in a controlled and more rigid academic way, has been neglected.” Nevertheless, the discovery of the therapeutic potency of spontaneity (Moreno, 2010) and its effect on developing human interactions has been central to the process of recognizing the importance of psychodrama. The relevance of our study is directly linked to the discussion of the effects of psychodrama in several different contexts of application, clinical and non-clinical (Kipper, 1978; D’Amato and Dean, 1988; Kipper and Hundal, 2003; Orkibi et al., 2017a,b; Azoulay and Orkibi, 2018; Testoni et al., 2018) and to the need for solid methodologies that gather mixed approaches and acknowledge the complexity of this type of intervention, not always easily amenable to empirical research (Kim, 2003). All in all, as stated by Kipper and Ritchie (2003, p. 23) in their meta-analysis about the effectiveness of psychodramatic techniques, “the findings appear to shed a positive light on the issue of the validity of psychodramatic interventions and to encourage research regarding the specific psychotherapeutic effects of its basic techniques.”

## Materials and Methods

This work is the result of a 5-year period of data collecting in a university clinic. The therapeutic setting was the typical psychodramatic one, with weekly sessions of around 2 h, in a group context with four to seven participants and two therapists in each session.

We will present the data in the following two ways: first, the group data as a whole and second, a single case following the HSCED rationale, as presented above.

### Participants

The participants were invited to enroll in this investigation and informed about their rights and what was expected from them in terms of the estimated amount of time and data to be collected. Informed voluntary consent forms were signed by every volunteer. During the research period, some of the group participants did not take part in the research.

These participants were doing therapy in a psychodrama group, and during the research two other therapists (auxiliary egos) were part of the team at different moments. All therapists had training in psychodrama, and the director is a trainer at the Portuguese Society of Psychodrama and is one of the researchers and author of this paper. Supervision of the treatment was done periodically by training members of the same society.

The five participants in this study (several volunteers were not included because the data produced was not considered enough to perform the analysis) were three women (the youngest was 27 years when beginning treatment and the oldest was 48 years old) and two men (33–35 years old). All the participants had university degrees and all were professionally active at the moment they began therapy.

### Procedure

Participants were invited for one, in some cases two, session(s) of data collecting, previous to the beginning of therapy. All these sessions were conducted by an independent researcher, a clinical psychologist that was not part of the therapeutic team. Clinical Outcomes in Routine Evaluation Outcome Measure (CORE-OM) and Revised Spontaneity Assessment Inventory (SAI-R) forms were filled by the participant, and the PQ interview protocol was used for constructing this idiosyncratic individual assessment instrument. On a weekly basis, the PQ was delivered to the participants prior to the session, and the Helpful Aspects of Therapy forms were sent via email after the session. The other outcome measures (SAI-R and CORE-OM) were filled in intervals no shorter than 6 months, depending on the availability of the participants. In these assessment moments, a CCI was conducted to assess the participant’s perspective of the process. At the end of therapy, an assessment session was held, and, whenever possible, a follow-up would take place 6 months after the end of treatment. The research protocol was approved by the Board of the clinic where the study took part.

### Measures

The different instruments used in this research were selected due to their pertinence to measure what we proposed, their robustness, their availability in the Portuguese language, and their international frequent use.

Clinical Outcomes in Routine Evaluation Outcome Measure (Evans et al., 2000, 2002; Lyne et al., 2006; Portuguese version by Sales et al., 2012) assesses the effectiveness of the clinical intervention and consists of 34 statements that the patients must evaluate on a five-point Likert scale, based on how frequently they experienced a certain mood during the previous week, in accordance with the following gradation: 0, “Not at all”; 1, “only occasionally”; 2, “occasionally”; 3, “often”; and 4, “very often or always.” This instrument is divided into four domains: Subjective Well-being (four items), Problems/Symptoms (12 items), Life Functioning (12 items), and Risk/Harm (six items). Of the 34 items, approximately 50% relate to problems of low intensity, such as “I felt tense, anxious, and nervous,” while the remaining 50% of items relate to problems of high intensity, such as “I felt panic or terror.” Twenty-five percent of all items concern positive statements with reverse scores. The level of psychological distress is quantified by the total score of the test (higher scores indicate more serious problems). Cronbach’s alpha coefficient for the 34 items was 0.89 and for the subscales of this measure were, respectively, Subjective Well-being (0.76), Problems/Symptoms (0.87), Life Functioning (0.85), and Risk/Harm (0.77), indicating good internal consistency reliability. The minimum score that can be achieved is 0 and the maximum 136.

Revised Spontaneity Assessment Inventory is a scale designed to measure spontaneity (Kipper and Shemer, 2006; work in progress Portuguese version by Gonzalez, 2012 and Martins and Gonzalez, 2018). Studies have shown this scale to be positively correlated with various dimensions linked with well-being, and negatively related to measures connected to pathological functioning, thus giving empirical support to Moreno’s (1953) thesis of a positive relationship between spontaneity, creativity, and health (Kipper and Shemer, 2006; Kipper et al., 2010; Gonzalez, 2012; Testoni et al., 2016). The SAI-R questionnaire, like the original SAI (Kipper and Hundal, 2005; Christoforou and Kipper, 2006) asks one initial question: “How strongly do you have these feelings and thoughts during a typical day?.” The question is followed by a list of 18 items describing different feelings and thoughts, such as “creative,” “happy,” “excited,” “uninhibited,” “satisfied,” and “do anything within the limits.” The participants are asked to respond using a five-point Likert scale, ranging from 1 (very weak) to 5 (very strong). Cronbach’s alpha coefficient was 0.92, indicating excellent internal consistency reliability. The minimum score that can be achieved is 0 and the maximum 90.

Personal questionnaire is an expanded target complaint measure, individualized for each client (Elliott et al., 1999, 2016; Portuguese version by Sales and Alves, 2016). It is generated from the PQ Problem Description Form, completed by the client during the screening process. It intended to be a list of problems that the client wishes to work on in therapy, stated in the client’s own words. Usually before each session, the participants were invited to fill his/her form, in which each sentence/problem should be rated on a seven-point Likert scale, corresponding to the way that issue was considered during the previous week, with seven being equivalent to maximum disturbance. PQ data meet criteria for evidence-based, norm-referenced measurement of client psychological distress for supporting psychotherapy practice and research.

Helpful aspects of therapy (HAT) is a post-session qualitative self-report instrument developed by Llewelyn (1988) that gathers information about the client’s perception of helpful and hindering events in psychotherapy (Castonguay et al., 2010; Sales and Alves, 2016; Portuguese version by Sales et al., 2007). In this instrument, participants are asked to describe particular and important aspects of the previous session and to rate how helpful or hindering these events were on a one-to-five-Likert scale (with one indicating *not hindering*/*helpful at all* and five indicating *extremely hindering*/*helpful*). Filling out the HAT becomes a routine part of the client’s overall therapy experience and appears to help clients process their therapy more effectively. The most common problems appear to be responses that are very brief, vague, or global.

Client Change Interview (Rodgers and Elliott, 2015) is a semi-structured interview that lasts, in average, between 30 and 45 min and can be performed by a third party every 8–10 sessions or at different intervals, at the end of therapy, and at follow-up. Using the PQ as a base, the interviewer asks for descriptions of their attributions for perceived changes, including helpful aspects of their therapy (information on negative aspects of therapy, medication, and other sensible clinical information is also collected). For each change identified, the client was asked to answer three questions about how surprising change was, if the change would have occurred without therapy, and how important the change was. A Likert scale of 1-to-5 was used for the answers.

### Data Analysis

To generate the group results, a quantitative variation analysis was performed following an HSCED-based approach. To compute the data, SPSS, version 24.0 for Windows was used (SPSS 24 Inc., Chicago, IL, United States).

To assess the evolution of the group, we carried out an in-group variation analysis for SAI-R, PQ, and CORE scales. First, the data were coded into overall scores of the scales. Second, CORE responses were categorized into more particular dimensions labeled as “Subjective Well-being,” Problems/Symptoms,” “Life functioning,” and “Risk/Harm,” as recommended in the literature (e.g., Lyne et al., 2006). Finally, we calculated mean intensity both for overall scale scores and for sub-scales. For the five participants, we could choose four different time points of evaluation, that correspond to different treatment spans for each of them. In general, the span between these time points was around 6 months (as said before, the data collecting times were chose individually and depending on the personal availability of the participants), so the total span corresponds to approximately 2 years of treatment. In only one of these cases does Time 4 correspond to the end of treatment.

Using the HSCED (Elliott, 2002) the single case received both quantitative and qualitative data analysis. For the quantitative analysis, we used the rationale proposed by Evans et al. (1998). This being said, we carried out the descriptive statistics (mean and standard deviation) for each scale, i.e., SAI-R, CORE, and PQ at several moments. For the CCI, we calculated the frequency for each one of the dimensions. For measuring the clinically significant changes, we compared the baseline (beginning), the end of therapy, and the 6-month follow-up values with the cutoff points. This cutoff (reported as caseness in **Table 1**) allows for determining whether a client is clinically distressed (if above cutoff) or not (if below of cutoff). The publication of Portuguese values for cutoff points is still in its last phase, so we are referring to the English ones, which we know, personally from the authors, to be quite similar to theirs. This cutoff points are available on the CORE System Handbook (Core System Group, 1998). Finally, to determine the reliability of changes, we calculated two differences – before vs. after treatment and before vs. 6-month follow-up – and compared them to the reliable change (RC) index, a measure of the variation based on the standard error (SE) of the measurement which takes account of two measurements (pre/post). There are several ways of calculating the criteria for both RC and clinically significant change. The ones we used are also summarized within the CORE System Handbook (Core System Group, 1998).

**Table 1:** John’s outcome data.

Scale	Caseness	RC min^∗^	Pre	Post	Pre–post difference	6-Month follow-up	Pre 6-month difference
CORE all items	1.19	0.72	1.59	0.58	1.01^a^	0.53	1.06^a^
All non-Risk	1.36	0.77	1.82	0.68	1.14^a^	0.61	1.21^a^
Subjective Well-being	1.37	0.90	2	1	1^a^	1.25	0.75
Problems/Symptoms	1.44	0.85	1.67	0.67	1^a^	0.67	1^a^
Life functioning	1.29	0.80	1.92	0.58	1.34^a^	0.33	1.59^a^
Risk/Harm	0.43	0.69	0.50	0.17	0.33	0.17	0.33
SAI-R	63	8.00	42	71	-29^a^	70	-28^a^
			**Pre**	**30M**	**Diff.**		
Personal questionnaire	3.00	0.53	4.67	2.13	2.54^a^	–	–

*Caseness, cutoff for determining whether client is clinically distressed; RC min, minimum value required change at p < 0.2; sources for values given: SAI-R, Spontaneity Assessment Inventory-Revised; CORE, Core System Group (1998); Personal Questionnaire (Barkham et al., 1996); M, months in treatment. ^a^Reliable improvement from therapy. ^∗^p < 0.02.*

## Findings

### Group Findings

**FIGURE 1:**
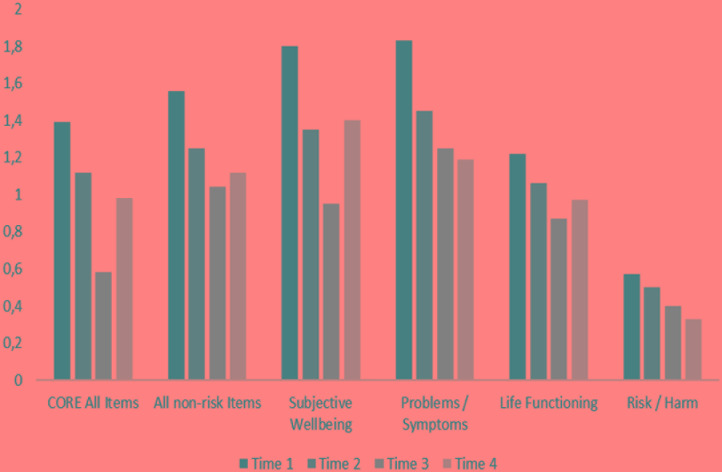
Group CORE means of scores over time.

As can be seen in **Figure 1**, all CORE values show a tendency to diminish during the course of treatment (T1 is at the beginning and T4 is after approximately 2 years of therapy), corresponding to a progress of the group. CORE’s sub-scales of Problems/Symptoms and Risk consistently diminished over time, while the other four diminished from the beginning until Time 3 and showed an increase from Time 3 to Time 4.

**FIGURE 2:**
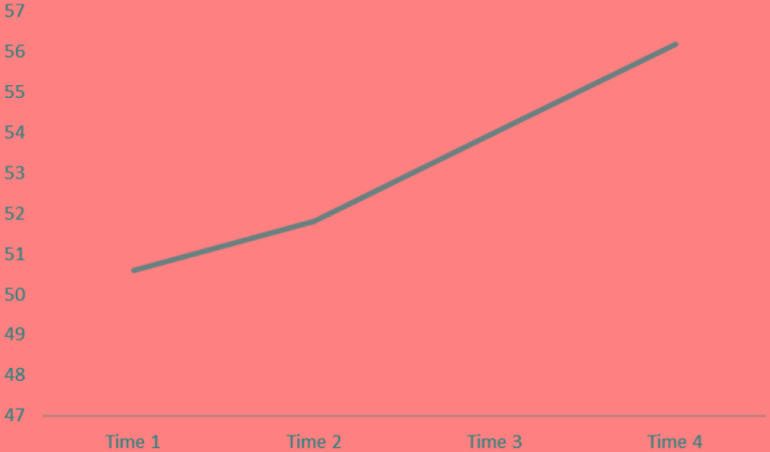
Group SAI-R scores over time.

The interpretation of the spontaneity line behavior should be made in an inverse way: the higher the results, the better the spontaneity. The spontaneity level of the group as a whole increased consistently throughout time, as can be seen in **Figure 2**.

**FIGURE 3:**
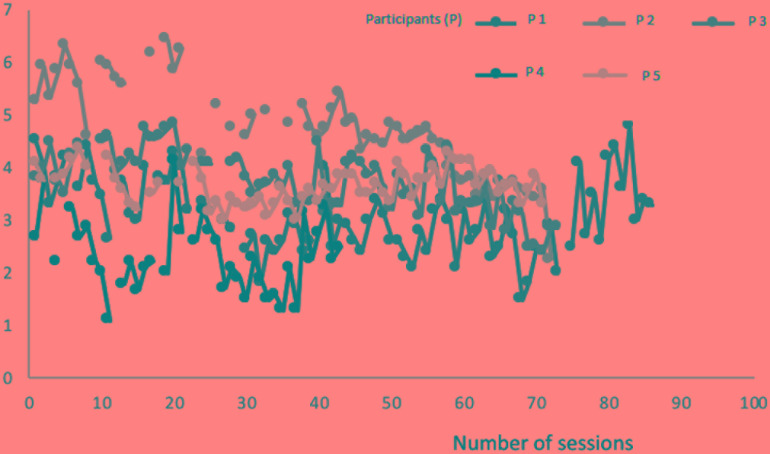
Group PQ means of scores over time.

In **Figure 3**, the PQ means of the five subjects across sessions can be seen, showing a decreasing tendency over time.

### Single Case Findings

In the presentation of this case, we shall follow the HSCED rationale previously presented, and will choose a participant (that will be addressed as John), based on the fact that he was the most regular of all group members in terms of responding to the set of research measures and that it was possible to do a 6-month follow-up assessment after he finished therapy. His therapeutic process lasted 5 years.

John contacted the group director weeks before his 35th birthday in order to start psychodramatic therapy, motivated by a pervasive personal crisis which he could not solve with previous psychotherapeutic processes. He had married 5 years before, but during the weeks after marriage two impactful events occurred: the suicide of his and his wife’s best friend and his cancer diagnosis. He began a successful oncological treatment and after this critical period he started to feel professionally unmotivated. After the birth of his first son, this personal crisis increased, affecting his marriage and his relationship to his parents and his only brother. He decided to go on a journey to South America and when he returned it became very difficult for him to continue his professional activity as a lawyer.

#### Outcome Measures

##### Personal questionnaire, CORE, and spontaneity assessment inventory

In his first interview for assessment purposes, the items he created for his PQ were the following (by order of importance):

(1) I feel depressed; (2) I am selfish; (3) my self-confidence is low; (4) I feel lost in professional terms; (5) It is hard for me to express emotions; (6) decision-making is difficult for me; (7) I tend to somatize; (8) I have a fusional relationship with my mother; (9) it is difficult for me to take paths that cause suffering; (10) I have a distant relationship with my father; (11) I have narcissistic features; and (12) I feel I am living a late adolescence.

Over time, near the end of treatment, he eliminated some items no longer considered as causing him suffering (ex: numbers 2, 5, 7, and 8) and brought in new ones (none of these changes in PQ will be taken into consideration in our graphic analysis). We will take a closer look at the evolution of the first six items, the ones most valued by John.

John’s first assessment on SAI-R and CORE revealed major distress and personal suffering. His spontaneity score (42) was significantly below the mean score for Portuguese men (63), and his CORE results can be considered clinical in all sub-scales, with life functioning values being the most critical and the Risk scale reaching worrying values. In the following graphics, John’s evolution over time can be seen.

**FIGURE 4:**
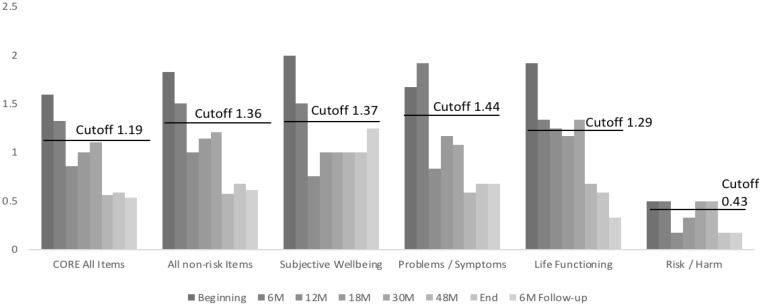
John’s evolution in CORE scales over time. M, months in treatment.

**FIGURE 5:**
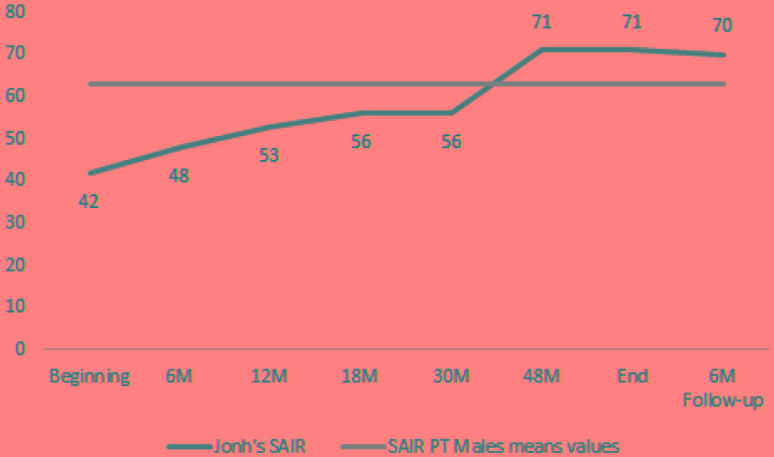
John’s SAI-R scores and Portuguese men mean. M, months in treatment.

Both his CORE (**Figure 4**) and spontaneity (**Figure 5**) values show consistent and significant improvements over time. In the case of CORE, from beginning to end he goes from above cutoff results to below cutoff ones. In the case of spontaneity as assessed by SAI-R, he starts in a remarkably low value, to finish above the mean for Portuguese men.

**FIGURE 6:**
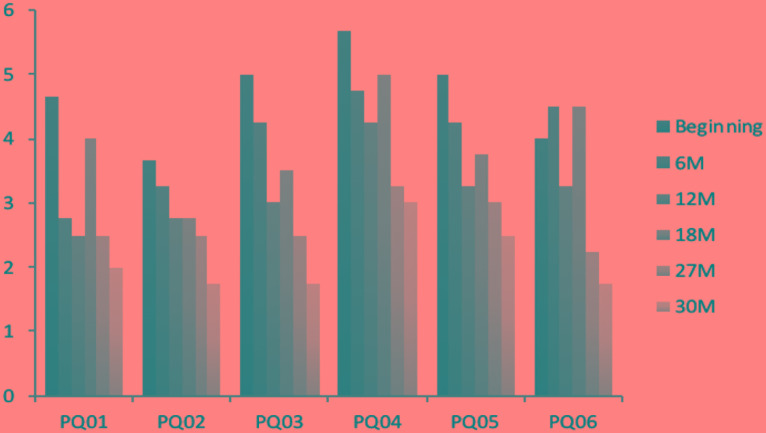
John’s means (four sessions) of PQ items values over time. M, months in treatment.

To facilitate the understanding of the PQ evolution throughout the first 30 months (we do not have access to the PQ data relating to the end of therapy), we distributed sessions in groups of four and calculated the mean for each one of these groups, attempting to thus characterize in the most reliable way John’s answers over a certain period of time. This allowed us to obtain mean scores for each item analyzed throughout six progressive time points (reported as Time in **Table 1**) of the therapeutic process, which are shown in **Figure 6**. As can be seen, the distress attributed to the main six items of his PQ decreases throughout time.

These results can be seen in **Table 1** that can help us with criteria that allow us to decide if observable changes in results can be considered. Based on the results in **Table 1**, we can see that all of John’s results in his first CORE assessment were above cutoff values, confirming his self-evaluation during the first contact with the therapist and the information given during the first interview with the researcher for the construction of his PQ. Furthermore, his spontaneity was significantly below the Portuguese mean for men (*M* = 62.96; *SD* = 10.21). The mean of his first four PQs also shows significant distress.

At the end of therapy, all results on his CORE (general and sub-scales scores) had gone below cutoff points. All these changes, except in the Risk subscale, can be considered reliable and clinically significant, as the difference between the values at the beginning and at the end is bigger than the minimum Required Change value. In follow-up, all these values are maintained below cutoff points, but the change in the Well-being subscale does not reach the 0.90 value needed to be considered significant.

Differences in SAI-R results are reliable and significant, and the same can be said about PQ results.

#### Process Measures

##### Helpful aspects of therapy (HAT)

John was the group participant who more frequently filled the HAT (87 forms). 160 events were described, 156 considered “helpful,” and 4 “not helpful” or “hindering” (not being chosen to be protagonist in one specific session, unpleasant feelings about another participant in two different occasions, and the departure of a group member).

The mean rating for the aspects considered as helpful was 4.1, ranging from 2.75 to 5. The first time John rated an event on the higher point of the scale was after 5 months of therapy. In his HAT, he states that “the dramatization of my cancer and the revisiting of the emotions felt during that moment of my life were very important. It was very important to feel emotions similar to the ones I experienced when I was ill, to share them with the group and to express them during the dramatization.”

Somehow the session of the previous week could have been a warm-up for this one, as he rated with a 4.75 in the helpful scale the way the group reacted to the theme – sexuality – that he addressed on that day. That session was, in fact, a turning point in group cohesion, as stated by several members.

The second time he considered an event in a session of maximum helpfulness was 2 months later, with the sharing of another participant about his mother’s suicide attempt, that made John contact with childhood memories of his own mother’s depressive phases.

Several other high evaluations of helpfulness occurred in the following weeks, but only 2 months later did he rate a session with another “5,” referring to a session during Christmas season in which the psychodramatic technique known as “Magic Shop” was used (Barbour, 1992). In John’s words:

“Yesterday’s session was amazing; during and after it, I felt a very strong positive energy that, beyond any doubt, is an energy from the group. After the warm-up, the true Christmas present arrived: the climb up the Magic Mountain (reminds me of Thomas Mann) to the Magic Shop. It was during the “way up” that I immediately felt something special, that made me say later on that any temptation of missing the session that night had absolutely dissipated. The dramatization was a great present from the therapists, because they received us with open arms, and allowed us to go away much lighter, closer to ourselves. All this created a wave of great opening and sharing, that resulted in a very strong tuning by the end of the session, when we shared what we asked for and what we received. We left this session with a full heart! I think that, from now on, we will have an even more cohesive group, even if Marcia was not here.”

In the same HAT form, he rates two other events as being very helpful (4.75): the reflection during the sharing phase about his sometimes overwhelming self-expectancies and about his difficulty opening up to others. Another aspect, rated 4.25, concerning the same session, relates to the feeling of closeness, trust, and empathy toward the therapeutic team. He particularly states that the fact that the group director was a participant in the dramatization (several versions of “The Magic Shop” technique include the director in the role of “shop owner”) was a “first time,” “surprising,” and “rare event,” felt as “contagious generosity.”

In **Table 2**, the events considered as helpful by John are categorized using the HAMPCAS system (Cruz et al., 2016).

**Table 2:** Type of events considered helpful.

Kind of event	*n*	%
Sharing by other members	52	24
Dramatizations by other members	44	20
His own sharing	41	18.5
His own dramatization	24	11
Sharing/comments by therapists	22	10
Role reversal technique	7	3
Group games	6	2.5
Social atom technique	4	2
Sculpture technique	4	2
Other techniques and events	16	7
Total	220	100

##### Client Change Interview (CCI)

Seven CCIs were conducted with John. The transcriptions are long, as some of the interviews lasted more than 1 h. Organized data from the interviews will be presented, and some examples given. For each change identified by the participant, three questions and rating scales (1–5) would be presented: “How much was the change expected?” (1, “Very much expected”; 5, “Very much surprised by it”); “How likely do you think it would have happened if you hadn’t been in therapy?” (1, “Very unlikely”; 5, “Very likely”); and “How important or significant to you personally do you consider this change to be?” (1, “Not important at all”; 5, “Extremely important”). In other words, in terms of change perception, the best answer from a participant would be the sequence of ratings 5–1–5 to these three questions.

Pertinent information about competing explanations for changes was collected. During the therapeutic process, John was not taking any medication. Supported and challenged by the group, he started several other activities, started to practice more sport, and, in the last period of his process, he joined a Biodanza group on a weekly basis. Fifty-three changes were identified, with 60% of the changes being “somehow surprising” (rated 4 on a 1–to–5 scale). Forty-four percent of them were considered by John as “probably not occurring” without therapy, and 9.5% “unlikely to occur.” Fifty-nine percent was considered “Very important” and 19% “Extremely important.” In **Table 3**, we can see a summary of these self-attributed changes.

**Table 3:** Change along therapy as rated by John in Client Change Interviews.

Changes (Total = 53) (values are reported as means)	Change was 1 – expected 3 – neither 5 – surprising	Without therapy 1 –unlikely 3 – neither 5 – likely	Importance 1 – not at all 3 – moderately 5 – extremely	Client change index
Time 1 (7 changes)	3.3	3.6	3.3	3
Time 2 (9 changes)	3.2	2.4	3.7	4.5
Time 3 (8 changes)	3.5	2.75	3.7	4.45
Time 4 (9 changes)	3.7	2.7	4.1	5.1
End (11 changes)	3.9	2.2	4.3	6
Follow-up (9 changes)	3.7	1.9	4.3	6.1

We performed a calculation of a value we called Client Change Index, by adding up the ratings of expectancy of change and its importance and subtracting the likelihood of change without therapy. On the last column of **Table 3**, we can see that this Index consistently increases in John’s self-rated therapeutic path. In order to have a specific criterion to choose the most significant changes from John’s CCIs, we selected the ones that had a Client Change Index higher than or equal to 7. From the 53 changes, 9 reached that criterion (**Table 4**).

**Table 4:** Most important changes stated in John’s CCIs.

Time	Change	Change was 1 – expected 3 – neither 5 – surprising	Without therapy 1 – unlikely 3 – neither 5 – likely	Importance 1 – not at all 3 – moderately 5 – extremely
Time 2	I am leading a healthier and more active life	4	2	5
Time 4	I am more flexible and open	4	2	5
End	I feel more serene	4	2	5
End	I no longer feel hostage to my friends	5	2	5
End	I can accept my parents exactly as they are	4	1	4
End	I can cope with mourning	5	1	5
End	I have grown closer to my mother	5	2	4
Follow-up	I am in a new relationship	5	2	5
Follow-up	I have a feeling of satisfaction and peace	4	2	5

These self-reported changes can be considered clinically pertinent issues, and the fact that the number of significant (according to the Client Change Index criterion) changes is bigger at the end of treatment and in follow-up is to be taken into consideration.

## Discussion

After presenting group and individual results from a complex and long process of data collecting, we shall reflect on the efficacy of these therapeutic processes, mostly based on the single case. Referring to the group as a whole, the scores lead us to the statement that, in general, progressively better results were shown in terms of spontaneity and the general and specific sub-scales of CORE-OM. Some of the CORE sub-scales showed an increase from the third to the fourth assessments. Consistent with these findings, **Figure 3** shows a general tendency for the means of the results obtained in the PQs of the members of the group to diminish, showing an improvement in the problematic situations they classified as important at the beginning of treatment.

These group findings are consistent with John’s case. Using recognized criteria to establish the reliability and clinical significance of changes in therapy (Barkham et al., 1996; Evans et al., 1998; Elliott, 2002) we can consider John’s improvements in CORE total and subscales scores (except Risk) from beginning to end of treatment as reliable and significant from a clinical point of view. In general, these changes were maintained in the 6-month follow-up.

His results with the spontaneity values go in the same direction. From the point of view of psychodramatic theory and practice, this is a particularly interesting finding, as Moreno strongly related individual spontaneity with general health and wellbeing (Moreno, 1953; Kipper and Shemer, 2006; Kipper et al., 2010; Gonzalez, 2012; Testoni et al., 2016). John’s PQ ratings showed a decrease during therapy. According to the same criteria used above, these changes can be considered clinically significant. Some of the items were personally removed by John for he no longer considered them significant.

The process/qualitative data written by John offer us a significant and fertile ground for reflections upon his therapeutic process. His first rating of an event as being extremely helpful (score = 5), just one session after feeling that his intimate sharing with the group about sexuality (an issue that was both important for the group, with several revelations made after his sharing, and to John, who felt totally welcomed) was very helpful too (4.75), occurred 5 months after the beginning of therapy. The therapeutic event was the dramatization of the moment he received his cancer diagnosis, when very strong feelings appeared, some of which had apparently been suppressed in order for him to cope with the treatment. He states that the possibility of dealing with these intense and overwhelming feelings and, especially, the possibility of sharing them with the other members of the group were particularly helpful.

Reading his HAT, we can identify several of the 14 therapeutic factors in groups that were suggested by Yalom and Leszcz (2005): acceptance/cohesion, self-disclosure, and self-understanding can be easily connected to his words, but existential factors, universality and catharsis, were present in this particular session too. It was a session where John was the protagonist, and the dramatization was directed to the representation of the different qualities of feelings and emotions connected to the memories of those overwhelming and probably poorly processed times. We believe there is a clear connection between these therapeutic decisions (choosing John to be the protagonist, the selected theme, and the general and specific psychodramatic techniques used) and his view of the helpful aspects of the session, including profiting from several, group therapy advantages.

It is important to stress the information presented in **Table 2**, showing the kind of events John considered helpful: 44% of the total 220 events underlined by him address the other members sharing and dramatizations. His own sharing and dramatizations sum up around 30% of the events considered helpful. This focus on the effect of group processes in his personal therapeutic process is reaffirmed in the other two extremely helpful events stressed by John: the impact he felt from the sharing of another group member, and the group dynamic that occurred on a session with the specific technique called “The Magic Shop.” He writes about “energy from the group,” “great opening and sharing,” “very strong tuning,” and the “even more cohesive group.”

John’s CCIs can help us in several ways. First of all, in excluding some alternative explanations for his changes, that we shall discuss further on. In John’s seven CCIs, 53 changes were identified. We calculated a Client Change Index that expresses the change expectancy, importance, and likeliness of occurrence without therapy. We find it significant that this index consistently increases during treatment, that is, throughout the 5 years in the group, John started to progressively give more value to his personal changes and attributing them to psychodrama.

## Conclusion

To conclude, we will follow the questions suggested by Elliott in his seminal paper on HSCED (Elliott, 2002) and try to use the indicated criteria in order to answer them. Among others, three questions are particularly important in the field of psychotherapy research: are participants on psychotherapy changing? Is therapy responsible for change? What in therapy is causing change?

Although we have reasons to suggest that there were changes in the group as a whole, we will focus our attention on the single case. Most of the direct evidences proposed by Elliott are met in John’s case: (a) he attributes several changes to therapy, stating that several of those important changes (**Table 4**) would probably not have occurred without it; (b) changes can be connected to therapy events (ex: the specific work on mourning and his statement that “I can cope with mourning,” or the repeated work, during dramatization, on helping him get in touch with his feelings, with other people’s feelings, through role reverse, and receive feedback from the other participants about how they experienced interacting with him, and his self-perceived change, expressed in his CCI, that “I am more flexible and open”); (c) clear and significant changes in symptoms and other indicators (CORE), in perceived suffering (PQ), and in variables positively associated with wellbeing (SAI-R) can be observed from beginning to end of therapy (**Table 1**); (d) furthermore, in several HATs it is possible to perceive that specific interventions made by the therapeutic team have a direct impact in John’s perception of the session as helpful (see the example of the session dedicated to staging the censored feelings associated with the cancer diagnosis during the dramatization).

It is simultaneously important to dismiss possible alternative explanations for changes. No pharmacological treatment was started or interrupted, no major diseases diagnosed or cured during treatment. John started to increase his physical activities while undergoing therapy, and in the last months of treatment he entered a group of Biodanza, a non-therapeutic, non-verbal expressive practice based on dance and bodily communication. Although these might be competing explanations for some of the changes, his decision to start these activities came as a consequence of his evolution during the first months of treatment, and with respect to Biodanza, he only started it very close to the end of therapy, when the main changes were already established. Some major life events occurred (birth of a second son, end of marriage) that had an impact on John’s general state, but the birth of his son did not directly influence the values of the main assessment results and the end of his marriage was part of the cause for some of the deterioration that made him extend his final phase of therapy. This period was not reflected in the main periodic assessments because it occurred shortly after the last evaluation (48 months), previous to the end of treatment. In any case, it was considered a very stressful event, whose negative impact was dealt with in therapy.

The main changes identified cannot be considered trivial or negative, as can be read from John’s words. Good reliability and clinical significance measures were used and several different measures utilized that can help to eliminate the effect of statistical artifacts in the explanation of change. Although relational and expectancy artifacts (pleasing the therapists/researchers and wishful thinking about own change) might have had some effect on these changes, their consistency, the multiple sources confirming them, and the frequently idiosyncratic language used by John to address them minimize that possibility.

Taking in consideration the arguments presented before, we believe we can affirm that John showed considerable and clinically reliable improvements during his time in the psychodrama group, and that therapy accounts for most of the changes. Although we can pinpoint some specific therapeutic techniques that were used and that are apparently connected to aspects valued by John as helpful, further research is needed to determine that connection.

Our last comment will concern the participation in the research as viewed by the group elements, by John in particular and by the therapeutic team. In several different interviews, John and others stated that being part of this research effort, filling the forms, in particular the HATs, and doing the CCIs became part of his/their therapeutic process, as moments for taking stock of the process. Regardless of the discussion about the possible therapeutic effects of being part of a research, which is not the aim of this work, there is no doubt that part of the hermeneutics associated with this kind of scientific research has common ground with therapeutic activity and that these participants become co-researchers of their own processes of change (see Elliott, 2002).

For the psychotherapeutic team, taking part on this research project was extremely challenging and fruitful. Receiving periodic feedbacks, from a weekly to a therapy-span basis, is an enriching experience for the psychotherapist, an opinion that goes along with the recent literature on feedback systems in psychotherapy (Reese et al., 2009; Lambert, 2013a,b; Wampold and Imel, 2015). The data collected and produced challenge the therapists/researchers to better understand the basics of their practice and to better connect it with theoretical issues, literature, and scientific methodology. It is our hope that this presentation of an experience where the roles of therapist and researcher meet will encourage others to join similar projects.

The “double hermeneutic” process referred by Elliott (2002), of a participant in therapy reflecting on his/her process of change and a researcher trying to track this path and interpret it, brought us to the creation of this paper. During the last phase of this long process, an idea took form: to contact John and ask him for a few lines about his long therapeutic process. Without the possibility of making him one of the signing authors of this work, for evident reasons, we wanted to thank him for all the energy invested in this journey. His answer to our request was the following paragraph:

“This psychotherapeutic process was one of my greatest life adventures. I quickly understood that the more I let myself go (as deeply and without filters as possible) the more respect, acceptance, and even admiration (and above all self-acknowledgment) I would receive from the group and therapists. The feeling of respect and admiration was reciprocal all along the process, of course. I was so lucky to feel loved and cared exactly for who I was and I took permission to express myself within this amazing group. Also, I had the privilege of witnessing the evolution of the other group members and, at the same time, the opportunity to strengthen the link of trust with the therapists. It was one of the most fertile and richest periods of my life and such learnings and experiences continue to flow deep within my heart.”

With his help and the help of the other participants and collaborators in this research project, we hope we could contribute to the study of the efficacy of psychodrama and, in doing so, to further validate the benefits of the profoundly human phenomenon that is the therapeutic encounter.

## Dedication

This work is dedicated to the memory of João Silva (1936–2018), a man of the theatre, director of GTT – Group of Therapeutic Theatre, founded by him in 1968 in the Júlio de Matos psychiatric hospital and one of the most proficuous and creative theatre groups of its kind. He gave visibility to and honored the therapeutic tradition of the theatre, during 50 years of Portugal’s sometimes turbulent recent history, and created a home for many that, throughout the years, shared their sufferings and joys on the stages built together with João. Influenced by the work of Moreno, he was himself a Man of Encounter.

## Ethics Statement

In this research it was clarified that participation was voluntary and that participants had the right to refuse it at any moment without penalty or prejudice to their interests. It was made clear that the names presented would be changed. All participants provided their written consent. The study was carried out in accordance with the recommendations presented in the Code of Ethics of the Portuguese Association of Psychologists, and the protocol approved by the Board of Clínica ISPA.

## Author Contributions

All authors listed have made a substantial, direct, and intellectual contribution to the work, and approved it for publication. The authors contributed equally to this manuscript. A-JG worked on the conceptualization, data collecting, analysis, and writing. PM worked with the analysis and writing. MdL worked with conceptualization, data collecting, and writing.

## Conflict of Interest Statement

The authors declare that the research was conducted in the absence of any commercial or financial relationships that could be construed as a potential conflict of interest.
